# The Use of Social Media for Health Promotion in Hispanic Populations: A Scoping Systematic Review

**DOI:** 10.2196/publichealth.5579

**Published:** 2016-07-11

**Authors:** Julia Hudnut-Beumler, Eli Po'e, Shari Barkin

**Affiliations:** ^1^ Vanderbilt University School of Medicine Nashville, TN United States; ^2^ Department of Pediatrics, Vanderbilt University School of Medicine Nashville, TN United States

**Keywords:** social media, social networking, Hispanic Americans, public health, health behavior

## Abstract

**Background:**

The Internet is an increasingly popular platform for public health interventions due to its distinct ability to communicate with, engage, and educate communities. Given the widespread use of the Internet, these interventions could be a means of equalizing access to information to address health disparities in minority populations, such as Hispanics. Hispanics are disproportionately affected by poor health outcomes, including obesity, diabetes, and human immunodeficiency virus/acquired immune deficiency syndrome. Although underserved and underrepresented, Hispanics are among the leading users of social media in the United States. Previous reviews have examined the use of social media in public health efforts, but, to our knowledge, none have focused on the Hispanic population.

**Objective:**

To conduct a scoping systematic review of the published literature to capture the ways social media has been used in health interventions aimed at Hispanic populations and identify gaps in existing knowledge to provide recommendations for future research.

**Methods:**

We performed a systematic review of the literature related to social media, public health, and Hispanics using the PubMed, PsycINFO, and EMBASE databases to locate peer-reviewed studies published between January 1, 2010, and December 31, 2015. Each article was reviewed for the following inclusion criteria: social media as a main component of study methodology or content; public health topic; majority Hispanic/Latino study population; English or Spanish language; and original research study. Relevant data were extracted from articles meeting inclusion criteria including publication year, location, study design, social media platform, use of social media, target population, and public health topic.

**Results:**

Of the 267 articles retrieved, a total of 27 unique articles met inclusion criteria. All were published in 2012 or later. The most common study design was a cross-sectional survey, which was featured in 10 of the 27 (37%) articles. All articles used social media for at least one of the following three purposes: recruiting study participants (14 of 27, 52%), promoting health education (12 of 27, 44%), and/or describing social media users (12 of 27, 44%). All but one article used multiple social media platforms, though Facebook was by far the most popular appearing in 24 of the 27 (89%). A diverse array of Hispanic populations was targeted, and health topics featured. Of these, the most highly represented were articles on sexual health directed toward Latino men who have sex with men (12 of 27, 44%). Healthy eating and active living received the second greatest focus (4 of 27, 15%).

**Conclusions:**

Social media offers a potential accessible venue for health interventions aimed at Hispanics, a group at disproportionate risk for poor health outcomes. To date, most publications are descriptive in nature, with few indicating specific interventions and associated outcomes to improve health.

## Introduction

The Internet is becoming an increasingly popular platform for public health interventions [1]. Key components of public health services, as outlined by the Center for Disease Control and Prevention, include educating communities about health issues, linking individuals to personal health services, and conducting research to find new insights and innovative solutions to health problems [2]. Compared with traditional forms of communication, the Internet has distinct advantages when it comes to communicating with, engaging, and educating communities [3]. For many individuals, the Internet is already their primary source of health information [1], and thus, it represents an opportune method of communication for public health interventions. Not only do Internet interventions have a high potential reach, but they have other advantages as well. Internet-based implementation allows individuals to access the intervention at a time that is convenient for them. That intervention can be highly personalized, based on participant data, and engaging, based on interactive tools and graphically rich content [1]. Additional benefits of using the Internet include low cost and rapid transmission through a wide community [4].

Public health practitioners and researchers alike have taken note of the potential the Internet holds for health interventions. Given the widespread use of the Internet, these interventions could be a means of addressing health disparities. Gibbons et al wrote of “the potential to connect underserved and underrepresented populations to important health information resources and to build social support for those affected by health care issues” through the use of the Internet [5]. Among classically vulnerable populations, such as rural, low-income, and racial and ethnic minorities [6], Hispanics are notable in that they “own smartphones, go online from a mobile device, and use social networking sites (SNSs) at similar—and sometimes higher—rates than do other groups of Americans” [7]. With regard to Internet use, 81% of Hispanic adults in the United States were on the Web in 2015, which is similar to the figure for all American adults of 84% [8]. However, when it comes to social media sites, Hispanics are among the leading users–75% of Hispanic adult Internet users were on Facebook in 2015, compared with 70% of white and 67% of black adult Internet users [9]. Hispanics are the leading users of other social media platforms as well, including Twitter and Pinterest [9]. Given the affinity of the Hispanic community for social media sites, they would appear to hold great potential for health interventions in this population. Moreover, Hispanics are at disproportionate risk for poor health outcomes, such as obesity, diabetes, and human immunodeficiency virus (HIV)/acquired immune deficiency syndrome (AIDS) [10-12].

Social media generally refers to Internet sites that allow users to generate and share content, which reached mainstream prominence in the early 2000s [13,14]. Examples include blogs, wikis, SNSs (eg, Facebook and Twitter), and content communities (eg, YouTube) [2,15]. Most previously published systematic reviews of social media–driven public health interventions focus on a particular health topic such as weight management, healthy lifestyle behaviors, or smoking cessation [16-18]. In 2012, Capurro et al performed a systematic review to capture the ways SNSs had been used for public health research and practice. Although their review included all health topics, none of the articles included specifically mentioned or focused on Hispanics [3]. To our knowledge, no other review has been conducted to assess the ways social media has been used for public health promotion and research among the Hispanic population in the United States, even though this is one of the fastest growing demographics in America [19,20]. Given the popularity of social media sites among the Hispanic community, the use of social media sites for public health efforts in other populations, and the disproportionate poor health outcomes in this population [3,9-12], the goals of this review were to inform public health practitioners and researchers on the current state of knowledge in the field and highlight gaps in need of further study and research. In this review, Hispanic and Latino/a have been used interchangeably.

## Methods

### Literature Search

We conducted a literature search using 3 databases: PubMed, PsycINFO, and EMBASE. Our search terms included “Hispanic,” “Latino,” and “Latina,” “social media,” “social network,” “social networking,” and “social network site,” as well as specific SNS names. Because SNSs are constantly evolving and changing in popularity, we used Wikipedia’s list of SNSs current to the first date of search—December 1, 2015. Of the 209 sites listed, 4 generated additional results within our query. The others were eliminated from the search terms. Using this process, our PubMed search included: (“Social Media”[Mesh] OR “social media”[tiab] OR “social network”[tiab] OR “social networking”[tiab] OR “social network site”[tiab] OR “facebook”[tiab] OR “google+”[tiab] OR “myspace”[tiab] OR “youtube”[tiab]) AND (“Hispanic Americans”[Mesh] OR “Hispanic”[tiab] OR “Hispanics”[tiab] OR “Latino”[tiab] OR “Latina”[tiab] OR “Latinos”[tiab] OR “Latinas”[tiab]) AND (“Public Health”[Mesh] OR “public health”[tiab] OR “Health Behavior”[Mesh] OR “health behavior”[tiab]).

For the other 2 databases, the query was modified to fit their search specifications. From the articles identified, we conducted a manual search of the articles’ references for other relevant studies.

### Article Selection

Inclusion criteria included were (1) social media as a main component of study methodology or content; (2) public health topic (ie, disease prevention or health promotion efforts); (3) majority Hispanic/Latino study population (ie, ≥ 50%); (4) published between January 1, 2010, and December 31, 2015; and (5) original research study.

We restricted our search to articles published in the last 5 years given the rapidly changing nature and popularity of Internet-based technology. We excluded studies not published in English or Spanish and duplicate articles. Two reviewers independently assessed each article based on these inclusion and exclusion criteria.

### Data Extraction

After identifying studies that met inclusion criteria, the following data were extracted from each article by 2 independent reviewers: publication year, geographic location, study design, sample size, study purpose, social media type (eg, Facebook, Twitter, YouTube, and so forth), social media use (eg, recruiting study participants, promoting public health messaging, and so forth), target population (eg, age, gender, sexual orientation, and so forth), and public health topic. Data were recorded in 2 unique spreadsheets. Based on observed trends, qualitative data syntheses were performed. An inductive approach was taken to data abstraction in which specific observations were then broadened to general conclusions.

## Results

### Articles Retrieved

We conducted our literature search in December 2015. After our initial search on December 1, we performed weekly searches of all 3 databases to identify any newly published articles. Our final search was on December 31. Both of the reviewers used the same set of searches performed and saved during this period to conduct their independent reviews. A total of 267 articles were returned from the 3 databases: 149 from PubMed; 30 from PsycINFO; and 88 from EMBASE. Seventy-seven articles were found to be duplicates. For the remaining 190 unique articles, we reviewed the title and abstract for the selection criteria. If necessary, we also reviewed the full text of the article. Between the 2 reviewers, there was an agreement of 97% regarding which articles met inclusion criteria. Any discrepancies in group allocation or data extraction were resolved through systematic review and discussion to arrive at a consensus. In total, 26 articles met inclusion criteria. The references of these articles were then scanned, and one additional article identified was found to meet inclusion criteria. Thus, a total of 27 articles were included in the final review. See Table 1 for a brief description of each of the articles included.

**Table 1:** Brief description of articles meeting inclusion criteria.

Author/year	Description	Social media site(s) used	Health topic
Graham 2012 [21]	Investigate efficacy of Web-based advertising for Spanish language smoking cessation website	MySpace Latino, MiGente, Website	Smoking cessation
Jaganath 2012 [22]	Describe the creation of a new, Facebook-based training curriculum for community leaders on HIV^a^ prevention	Facebook, MySpace	Sexual health
Justice-Gardiner 2012 [23]	Compare a traditional media outreach campaign with a new media outreach campaign aimed at Hispanic cancer survivors and their families	Facebook, Twitter, Website	Cancer
Vyas 2012 [24]	Examine use of SMS^b^ and social media for decreasing sexual risk taking among Latino youth	SMS, Facebook, YoutTube, Twitter, MySpace	Sexual health
Young 2012[25]	Determine whether peer leaders can be recruited for a community-based health intervention using social media	Facebook, MySpace, Craigslist	Sexual health
Dixon-Gray 2013 [26]	Develop and implement a social marketing campaign to increase preconception health knowledge among second-generation Latinas	Facebook, MySpace, Website	Women’s health
Landry 2013 [27]	Examine associations between new media use and sexual behaviors	SMS, unspecified social media	Sexual health
Tucker 2013 [28]	Examine the association between exposure to alcohol or other drug-related media and use of alcohol among adolescents	Facebook, MySpace	Substance abuse
Young 2013 [29]	Determine the feasibility and acceptability of using SNSs^c^ to facilitate HIV-related discussions and requests for home-based HIV test kits in Latino MSMs^d^	Facebook, MySpace, Craigslist	Sexual health
Young 2013 [30]	Understand the relationship between social media sex-seeking and sexual risk behaviors in Latino MSMs	Facebook, MySpace, Twitter, Grindr, Adam4Adam, Manhunt	Sexual health
Young 2013 [31]	Determine the feasibility of recruiting peer leaders for a community-based health intervention using social media	Facebook, MySpace	Sexual health
Young 2013 [32]	Explore the feasibility of recruiting minority MSM Facebook users for HIV prevention studies	Facebook Craigslist, Website	Sexual health
Young 2013 [33]	Determine whether social networking communities can increase HIV testing in Latino MSMs	Facebook, Craigslist, Website	Sexual health
Young 2013 [34]	Explore associations between stimulant use, sexual risk behaviors, and social networking among Latino MSMs	Facebook, Craigslist	Substance abuse
Barrera 2014 [35]	Examine comparative impact of keywords in an Web-based campaign to recruit pregnant Latina women to an Internet intervention	Website	Women’s health
Ferguson 2014 [36]	Examine television, social media, and peer competition influences on body dissatisfaction and eating disorder symptoms in Hispanic adolescent females	Facebook, Twitter, YouTube, Wordpress, multiplayer online gaming sites	Body image and eating disorders
Hanson 2014 [37]	Determine use of social media for health care–related purposes among medically underserved primary care patients	Facebook, MySpace, LinkedIn, YouTube, Twitter, blogs, SMS texting	Patient-provider communication
Martinez 2014 [38]	Recruit Spanish-speaking, Latino gay couples with social media to an HIV prevention study	Facebook, Craigslist, Grindr, SCRUFF, Jack’d, Instagram, SMS, Website	Sexual health
Quintililani 2014 [39]	Improve weight, diet, and physical activity among low socioeconomic status public housing residents with social media campaign component to intervention	SMS, Facebook	Healthy eating and active living
Young 2014 [40]	Describe study retention among Latino MSMs 1 year after a 12-week, social networking–based HIV prevention trial	Facebook, MySpace, Craigslist	Sexual health
Young 2014 [41]	Assess the feasibility and acceptability of using SNSs as a health research platform among Latino MSMs	Facebook, MySpace, Craigslist	Healthy eating and active living
Chiu 2015 [42]	Assess association between HIV status, SNS use, and sexual risk behaviors among Latino MSMs	Facebook, MySpace, Grindr, Orkut	Sexual health
Criss 2015 [43]	Explore how health information sources inform decision-making among Hispanic mothers	Facebook, YouTube, Website	Women’s health
Frerichs 2015 [44]	Assess readiness of community to address obesity and adopt healthy lifestyles following social media campaign	Facebook, YouTube, Website	Healthy eating and active living
Lee 2015 [45]	Examine the association between Web-based health information-seeking behaviors and health behaviors	Facebook, Twitter, MySpace, Website	Healthy eating and active living
Price 2015 [46]	Summarize public awareness activities on epilepsy to highlight communication channels	Facebook, Twitter, Website	Epilepsy
Smaldone 2015 [47]	Assess Latino parent and adolescent preferences in the use of mobile technologies and social media for provider-patient communication	Facebook, Twitter, MySpace, AIM, iChat, Skype, Oovoo, SMS	Patient-provider communication

^a^HIV, human immunodeficiency virus.

^b^SMS, short message service.

^c^SNS, social networking sites.

^d^MSM, men who have sex with men.

### Trends in Publication Dates and Study Locations

All articles meeting inclusion criteria were published in the last 4 years, from 2012 to 2015. In 2012, there were 5 articles published on the topic. That number increased to 9 in 2013, 7 in 2014, and 6 in 2015. No earlier articles retrieved in the search met inclusion criteria. With regard to study location, 26 of the 27 articles (96%) detailed studies that were conducted in the United States. The one remaining study was conducted internationally with most participants coming from Central and South America.

### Social Media Platforms

Of the social media platforms used, Facebook was by far the most common, appearing in 24 of the 27 articles (89%). Other popular platforms included MySpace (56%), websites (44%), Twitter (30%), Craigslist (26%), and YouTube (19%). Fourteen other social media platforms, such as Instagram, Grindr, MiGente, and LinkedIn, were mentioned in the articles, although with lesser frequency. All but one study used multiple social media platforms.

### Types of Studies

The most common study design was a cross-sectional survey, which was featured in 10 of the 27 articles (37%). Most surveys were used to capture social media usage, users’ characteristics, or users’ communication preferences. The next most common study designs were qualitative studies and randomized control trials. Qualitative studies appeared in 4 of the 27 articles (15%), and randomized control trials were featured in an equal number (4 of 27, 15%). Of note, 2 of the 4 papers featuring randomized control trials are from the same study, the Harnessing Online Peer Education study, meaning that in total there were only 2 unique randomized control trials identified in this review. Of the remaining studies, there were 3 (11%) prospective cohort studies, 3 (11%) mixed methods studies, and 3 (11%) reports on public health campaigns.

### Uses of Social Media

All of the articles included in this review used social media for at least one of the following 3 purposes: recruiting study participants, promoting health education, or describing users/usage characteristics. Most articles (14 of 27, 52%) used social media to identify and recruit study participants. However, using social media for health education (12 of 27, 44%) and describing the characteristics of social media users were both also highly represented in the articles (12 of 27, 44%). Though less common, a few studies assessed participants’ health communication preferences (3 of 27, 11%), specifically whether they were open to receiving health information, including general public health messages and personal messages from health care providers, via social media. Finally, in 2 of the articles (7%), social media was highlighted as a way to retain study participants. See Table 2 for uses of social media by target population and health topic.

**Table 2:** Uses of social media for health interventions by targeted Hispanic population and health topic.

		Uses of social media^a^				
		Recruiting study participants (n=14)	Promoting health education (n=12)	Describing users or usage characteristics (n=12)	Assessing communication preferences (n=3)	Retaining study participants (n=2)
Target Hispanic population^b^						
	Men who have sex with men (n=12)	12 (86%)	7 (58%)	4 (33%)	0 (0%)	1 (50%)
	Adolescents (n=5)	0 (0%)	0 (0%)	4 (33%)	2 (67%)	0 (0%)
	General population (n=3)	0 (0%)	2 (17%)	1 (8%)	0 (0%)	0 (0%)
	Pregnant women and young mothers (n=3)	1 (7%)	1 (8%)	1 (8%)	0 (0%)	0 (0%)
	Medically underserved (n=1)	0 (0%)	0 (0%)	2 (17%)	1 (33%)	0 (0%)
	Public housing residents (n=1)	0 (0%)	1 (8%)	0 (0%)	0 (0%)	1 (50%)
	Cancer survivors (n=1)	0 (0%)	1 (8%)	0 (0%)	0 (0%)	0 (0%)
	Smokers (n=1)	1 (7%)	0 (0%)	0 (0%)	0 (0%)	0 (0%)
Health topic^c^						
	Sexual health (n=12)	10 (71%)	6 (50%)	5 (42%)	1 (33%)	1 (50%)
	Healthy eating and active living (n=4)	1 (7%)	3 (25%)	1 (8%)	0 (0%)	1 (50%)
	Women’s health (n=3)	1 (7%)	1 (8%)	1 (8%)	0 (0%)	0 (0%)
	Substance abuse (n=3)	1 (7%)	0 (0%)	2 (17%)	0 (0%)	0 (0%)
	Patient-provider communication (n=2)	0 (0%)	0 (0%)	2 (17%)	2 (67%)	0 (0%)
	Body image and eating disorders (n=1)	0 (0%)	0 (0%)	1 (8%)	0 (0%)	0 (0%)
	Cancer (n=1)	0 (0%)	1 (8%)	0 (0%)	0 (0%)	0 (0%)
	Epilepsy (n=1)	0 (0%)	1 (8%)	0 (0%)	0 (0%)	0 (0%)
	Smoking cessation (n=1)	1 (7%)	0 (0%)	0 (0%)	0 (0%)	0 (0%)

^a^Articles may contain elements of multiple categories (n>27).

^b^Articles contain only one target Hispanic population (n=27).

^c^Articles contain only one health topic (n=27).

### Target Populations

With regard to target population, most articles focused on certain populations within the Hispanic community as opposed to individuals with specific medical conditions. An overwhelming number of the articles included in this review focused on Latino men who have sex with men (MSM). Forty-four percent (12 of 27) of articles targeted this population. However, 10 of these articles derived from a single study, the Harnessing Online Peer Education study. Other populations featured included adolescents (5 of 27, 19%), the medically underserved (1 of 27, 4%), public housing residents (1 of 27, 4%), and the general Hispanic population (3 of 27, 11%). Of these groups, the most commonly targeted were Hispanic adolescents and the general Hispanic population. Although less common, individuals with specific medical conditions were the focus of a few of the studies. These conditions included pregnant women and young mothers (3 of 27, 11%), cancer survivors (1 of 27, 4%), and smokers (1 of 27, 4%). Moreover, of the 14 articles that used social media to recruit study participants, 12 (86%) targeted MSM, followed distantly by pregnant women and young mothers (1 of 14, 7%) and smokers (1 of 14, 7%). None of the articles targeted at other populations used social media for recruitment. Of the various groups targeted, MSM were also the most highly represented with regard to articles promoting health education (7 of 12, 58%), describing user characteristics (4 of 12, 33%), and retaining study participants (1 of 2, 50%).

### Health Topics

Of the health topics featured in the included articles, sexual health was by far the most highly represented, appearing in 12 of the 27 articles (44%). Two of these articles related to sexual health and risk taking behaviors among Hispanic adolescents. The remaining 10 articles were focused on sexual risk behaviors and HIV prevention among Latino MSMs. Other health topics that featured prominently were healthy eating and active living (4 of 27, 15%), women’s health (3 of 27, 11%), and substance abuse (2 of 27, 7%). Additional topics discussed include patient-provider communication (2 of 27, 7%), body image and eating disorders (1 of 27; 4%), cancer (1 of 27; 4%), epilepsy (1 of 27; 4%), and smoking cessation (1 of 27; 4%). Of the health topics, sexual health featured prominently in articles using social media to recruit study participants (10 of 14, 71%), promote health education (6 of 12, 50%), describe user characteristics (5 of 12, 42%), and retain study participants (1 of 2, 50%).

## Discussion

### Principal Findings

Given Hispanic’s disproportionate risk for poor health outcomes and high affinity for social media, public health interventions that use social media may be a means of addressing the health disparities present in this community [5,9-12]. With no articles published before 2012 and 27 articles published in the last 4 years relating to the use of social media for health interventions in Hispanic populations, this is evidently a nascent field in the realm of public health for this population. Of the 27 articles, 24 related to research, whereas only 3 were reports on public health campaigns, suggesting that using social media for health interventions in Hispanics is still mostly in the descriptive phase of research. It is not yet a common part of public health practice or published research. Although almost all articles referenced studies that included Facebook (89%), most used multiple social media platforms or complemented social media with other relatively new forms of communication, such as SMS or email. This trend deviates from previous reviews in which most studies depended on a single social media platform [3,17]. While text messaging on its own has received support as a tool for behavior change in disease prevention and management, the effectiveness of Internet interventions has been enhanced when coupled with additional methods of communication, especially SMS [48-50]. These distinct platforms may engage different aspects of the behavior change process, resulting in increased effectiveness of the intervention as a whole [50]. The use of multiple communication platforms by most studies included in this review may be a product of these findings, which come from previous trials that have sought to use the Internet to address health problems in other populations.

Perhaps given the relatively young nature of the field, few articles included in this review used randomized control trials. Four of the 27 articles featured randomized control trials; however, 2 of these articles come from the same study, suggesting that the lack of rigorous trial data is even less than it initially appears. Randomized control trials may also be underrepresented because of an inherent or perceived difficulty of using this study design for social media–based trials. Barrera et al noted the challenge in striking the right balance between addressing participant expectations of a trial and developing a social media–based study recruitment tool that captured the scope of the intervention without being too burdensome for Web users to access [35]. Moreover, few social media sites offer the option for closed-access or private groups. On most sites, content is open and freely available to everyone, which does not allow for the creation of intervention and control groups as would be necessary in a randomized control trial. Facebook is one exception. Part of Facebook’s popularity in the articles included here may stem from the ability to create multiple private groups using the site, thus allowing for different exposure conditions.

Cross-sectional surveys and qualitative studies were the most highly represented study designs. These study designs align well with the way social media is currently being used for health interventions in the Hispanic population. While some studies incorporated social media in multiple ways, every article included in this review used social media for at least one of the following 3 purposes: (1) study recruitment; , (2) health education promotion; , or (3) description of users/usage characteristics. Cross-sectional surveys and qualitative studies are appropriate for such descriptive purposes. Given the current gaps in knowledge, future studies should be designed as randomized control trials to test the effectiveness of social media–based health education programs and interventions for behavior change in Hispanic populations. Of the studies that have already been conducted, there is evidence that social media can be effective in recruiting study participants [25,31,32,38], increasing health knowledge [26], encouraging participants to engage in health discussions [33], increasing rates of testing for sexually transmitted diseases [29,33], and promoting study retention [40]. Only 2 studies specifically cited the use of social media as a means of promoting retention among study participants. Because of the challenges in retaining human subjects in longitudinal studies, this area merits continued study.

With regard to target study populations, a preponderance of articles focus on Latino MSMs. Because many of these articles come out of a single study, the number of studies that use social media–driven health interventions in Latino MSMs is actually far fewer. Three unique studies feature Latino MSMs—a similar number to that for Hispanic adolescents, Latinas who are pregnant or young mothers, and the Hispanic population at large. With only a handful of studies focused on each of these populations and fewer still on other groups like the medically underserved, continued research is needed for all Hispanic populations to further explore the efficacy and potential of social media–driven health interventions. Particular attention should be directed at the medically underserved and economically disadvantaged as a potential way to address health disparities among Hispanics [5]. Due to the large number of articles focusing on social media–based HIV prevention tools in Latino MSMs, sexual health is highly represented among the health topics featured in this review. However, once again, the actual number of unique studies dealing with sexual health is far fewer such that roughly an equivalent number of studies have been conducted to examine the use of social media in sexual health, healthy eating and active living, and women’s health interventions. With only a few studies conducted on each topic, each of these merits further research, especially given the increased risk of poor health outcomes for Hispanic Americans compared with non-Hispanic, white Americans in these areas, specifically heightened risks for HIV/AIDS, obesity, diabetes, and late entry into or complete lack of prenatal care [10-12,51].

From the research that has been done, social media has been shown to be an accessible and effective method of engaging a variety of Hispanic populations in diverse health behavior change topics. Despite previous reports showing that Hispanics who are younger, more affluent, English-speaking, and native born are more likely to use social media [7-9], there is early evidence to suggest that social media can also be an effective platform for reaching other members of the Hispanic community who tend to fall outside of these categories, such as community health center patients [37]. For public health practitioners, social media holds great potential. There are already examples community-specific efforts, such as Saludable Omaha which used Facebook and YouTube to promote healthy eating and active living among Omaha’s Hispanic population [44], and nationwide campaigns, like that undertaken by the Epilepsy Foundation to increase public understanding and awareness of epilepsy among Hispanics through Facebook and Twitter [46]. Public health practitioners should both encourage the use of relevant pre-existing social media resources and develop new, social media–based public health campaigns for Hispanic populations.

### Limitations

A number of limitations do exist to this review. We limited our search to published, peer-reviewed journals, which could reflect a publication bias and does not include studies in progress but not yet published. In addition, we limited our search to articles published in the last 5 years, meaning that we may have missed articles published earlier. Given that all articles meeting inclusion criteria were published after 2012, this is unlikely. Moreover, because of the rapidly changing nature and popularity of Internet-based technology, what may have been relevant and effective over 5 years ago may be outdated today.

### Conclusions

Hispanics are at increased risk for poor health outcomes, such as obesity, diabetes, and HIV/AIDS. Given that Hispanics are among the leading users of social media in the United States, there is an opportunity to use this technology for health interventions. Although a relatively new field, use of social media is quickly growing and a diverse array of target populations and health topics have been studied. To date, most publications are descriptive in nature and the full potential of social media–driven interventions for affecting health behavior change has yet to be fully realized in the Hispanic population.

## Abbreviations

MSM: men who have sex with men
SMS: short message service, commonly referred to as “text messaging”
SNS: social networking site
